# Engineering of LiTaO_3_ Nanoparticles by Flame Spray Pyrolysis: Understanding *In Situ* Li-Incorporation into the Ta_2_O_5_ Lattice

**DOI:** 10.3390/nano14151257

**Published:** 2024-07-27

**Authors:** Pavlos Psathas, Areti Zindrou, Anastasia V. Spyrou, Yiannis Deligiannakis

**Affiliations:** Laboratory of Physical Chemistry of Materials & Environment, Department of Physics, University of Ioannina, 45110 Ioannina, Greece; pavlospsatha@gmail.com (P.P.); a.zindrou@uoi.gr (A.Z.); anast.spirou@yahoo.gr (A.V.S.)

**Keywords:** lithium tantalate, perovskite, flame spray pyrolysis, high-temperature particle residence time, H_2_, photocatalysis

## Abstract

Lithium tantalate (LiTaO_3_) perovskite finds wide use in pyroelectric detectors, optical waveguides and piezoelectric transducers, stemming from its good mechanical and chemical stability and optical transparency. Herein, we present a method for synthesis of LiTaO_3_ nanoparticles using a scalable Flame Spray Pyrolysis (FSP) technology, that allows the formation of LiTaO_3_ nanomaterials in a single step. Raman, XRD and TEM studies allow for comprehension of the formation mechanism of the LiTaO_3_ nanophases, with particular emphasis on the penetration of Li atoms into the Ta-oxide lattice. We show that, control of the High-Temperature Particle Residence Time (HTPRT) in the FSP flame, is the key-parameter that allows successful penetration of the -otherwise amorphous- Li phase into the Ta_2_O_5_ nanophase. In this way, via control of the HTPRT in the FSP process, we synthesized a series of nanostructured LiTaO_3_ particles of varying phase composition from {amorphous Li/Ta_2_O_5_/LiTaO_3_} to {pure LiTaO_3_, 15–25 nm}. Finally, the photophysical activity of the FSP-made LiTaO_3_ was validated for photocatalytic H_2_ production from H_2_O. These data are discussed in conjunction with the role of the phase composition of the LiTaO_3_ nanoparticles. More generally, the present work allows a better understanding of the mechanism of ABO_3_ perovskite formation that requires the incorporation of two cations, A and B, into the nanolattice.

## 1. Introduction

Perovskites are efficient materials in electrocatalytic applications due to their low activation energy and high electron transfer kinetics; they also have the potential to be used as efficient photocatalysts due to their long-term stability and selectivity in catalytic reactions [1,2]. In the family of perovskites, titanates, tantalates, niobates, vanadates and ferrites have been used in many applications such as gas sensors, solid oxide fuel cells, dielectrics, ferroelectrics and photocatalysts for water splitting, to name a few [3,4,5]. Tantalum pentaoxide (Ta_2_O_5_) and its alkali tantalate perovskites, like lithium-tantalate (LiTaO_3_), sodium-tantalate (NaTaO_3_) and potassium-tantalate (KTaO_3_), have been demonstrated as highly effective materials in photocatalytic water splitting and CO_2_ reduction [5,6,7], making them promising candidates for solar energy conversion applications. Their photocatalytic activity is both attributed to, and determined by, the *d*-orbitals of Ta located at the conduction band, as well as to the efficient carrier delocalization promoted by small distortions in TaO_6_ bonds [2,8]. 

Tantalate perovskites have been shown to be able to perform water splitting without the use of a cocatalyst [9,10] under proper UV photon excitation, i.e., their energy gap lies around 4.7 eV for LiTaO_3_, 4 eV for NaTaO_3_ and 3.6 eV for KTaO_3_ [10,11]. Among the alkali tantalates, LiTaO_3_ stands out as one of the most notable materials due to its remarkable physical properties, including piezoelectricity, pyroelectricity and ferroelectric characteristics [12]. Kato and Kudo investigated alkali tantalates and their photocatalytic water splitting ability, i.e., splitting water into H_2_ and O_2_ without the use of cocatalysts such as Pt or NiO [13,14].

So far, the methods used to synthesize perovskite are numerous, and all of them require high temperatures, i.e., sol–gel [15], hydrothermal [16], microwave [17,18] and flame aerosol synthesis [4,19,20]. The synthetic approach can have a great impact on the properties of the final material, affecting the morphology, shape, crystallite size, specific surface area (SSA) and structure of the perovskite. Compared to other synthetic methods, the fundamental advantage of flame spray pyrolysis (FSP), a flame aerosol synthesis route, stems from its straightforward scalability, enabling industrial production yields to reach kilograms per hour [21]. However, the synthesis of perovskite ABO_3_ nanoparticles using FSP is rather challenging considering that we demand the concomitant insertion of two cations, A and B, into the lattice of ABO_3_ at the correct stoichiometry of 1:1:3. 

So far, there are few works in the literature regarding the FSP synthesis of ABO_3_ nanostructures; for example, Angel et al. synthesized LaMO_3_ (M = Mn, Fe, Co) nanomaterials using FSP and investigated the effect of dispersion gas flow on both the particle size and morphology [22]. In another work, Yuan et al. used the FSP method to deposit CuO quantum dots onto SrTiO_3_ perovskite [23]. The authors explored the role of copper loading onto perovskites and optimized the catalytic efficiency for the complete oxidation of lean CO and CH_4_, with the best-performing nanocomposite being 15 mol% CuO. Our group used FSP to synthesize BiFeO_3_ perovskite or mullite-type Bi_2_Fe_4_O_9_, which proved to be both efficient in the catalytic reduction of 4-nitrophenol to 4-aminophenol [24] and a highly efficient photocatalyst for O_2_ production from H_2_O [25]. 

In general, FSP is an effective method for the production of particles well-below 100 nm, a characteristic that might be difficult to achieve using wet synthesis routes. However, the crystallinity and phase transformation of flame-made perovskites are not straightforward. Kho et al. investigated the degree of crystallinity of as-prepared FSP-made BiVO_4_ nanoparticles [26]. By varying the filter temperature (T_f_) during FSP synthesis, the authors transitioned from the initially dominant amorphous to highly crystalline monoclinic-scheelite BiVO_4_ when T_f_ was greater than 360 °C. The crystallization of BiVO_4_ is highly dependent on T_f_, suggesting that, the short residence time of the particles in the FSP flame, is inadequate to induce crystallization within the flame. The same trend was verified by our group in the case of FSP synthesis of W- and Zr-doped BiVO_4_ [27]. Recently, we showed that FSP can achieve the synthesis of highly crystalline NaTaO_3_ under the condition that a well-defined protocol is adopted [28]. Specifically, we found that in FSP-made NaTaO_3_, the combustion profile in the FSP flame, i.e., the precursor/dispersion ratio (P/D), has a significant impact on both the crystallinity and the phase composition of the final material. A higher P/D ratio, where P corresponds to a higher solvent feed flow in the flame that boosts combustion enthalpy, favors the formation of 100% crystalline NaTaO_3_ perovskite [28].

Herein, our aim was to develop an FSP process protocol to successfully synthesize crystalline LiTaO_3_ nanomaterials. To do this, we systematically studied the thermodynamics of the FSP process that control the transition from {amorphous Li} + {Ta_2_O_5_} to crystalline LiTaO_3_ perovskite. The underlying solid-state process involves the incorporation of Li atoms into the Ta_2_O_5_ lattice and thermodynamic stabilization to a stable LiTaO_3_ crystal. Thus, our specific aims were [i] to use FSP to synthesize a series of amorphous Li/Ta_2_O_5_/LiTaO_3_ nanomaterials with different percentages of the three components, [ii] to show that amorphous Li/Ta_2_O_5_ could be transformed to crystalline LiTaO_3_ in a single post-FSP calcination step, [iii] to optimize the FSP process configuration in order to obtain high-purity (>95%) LiTaO_3_ in one step and [iv] to validate the functional quality of the resulting LiTaO_3_ in photocatalytic H_2_ production from H_2_O. 

By an analysis of key data (XRD, Raman) with the aid of TEM, we provide a comprehensive physicochemical frame that allows an understanding of the Li/Ta_2_O_5_→LiTaO_3_ transition in the FSP flame. We show that the HTPRT is the key parameter that must be optimized via control of the FSP reactor parameters.

## 2. Materials and Methods

### 2.1. Synthesis of LiTaO_3_ Nanoparticles via Flame Spray Pyrolysis (FSP)

**Figure 1:**
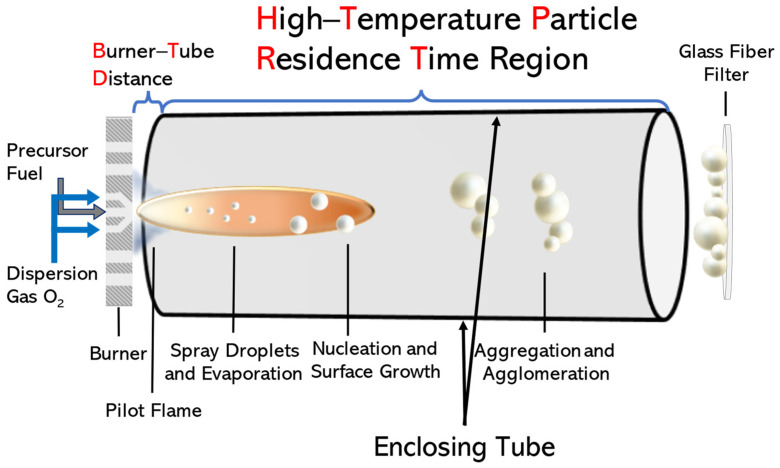
Schematic representation of the FSP process. The HTPRT signifies the region of the FSP process where the T-profile is high enough to allow phase evolution of the nanolattice, i.e., the Li/Ta_2_O_5_→LiTaO_3_ transition in the present case. We used a metal tube to enclose the flame, which provided a longer HRPRT. Adjustment of the BTD allows for control of ambient air entrainment, which acts as a flame coolant.

For the synthesis of LiTaO_3_ nanoparticles (NPs), for convenience, we codenamed the FSP-made LiTaO_3_ materials as LTO. A single-nozzle FSP reactor was used, schematically illustrated in Figure 1. Precursor solutions were prepared by dissolving 0.3 M Ta (V) n-Butoxide (99.9% Ta, STREM (Newburyport, MA, USA)) and 0.3 M Li-Butoxide (98%, STREM (Newburyport, MA, USA)) in xylene. Each precursor solution was then fed into the FSP system through a capillary with a steady precursor flow rate (P) of 3, 5, or 9 mL min^−1^ and dispersed into fine droplets using an oxygen dispersion flow rate (D) of 9, 5, or 3 L min^−1^ (Linde 99.999%). To initiate combustion, a premixed O_2_/CH_4_ pilot flame at flow rates of 4 L min^−1^ and 2 L min^−1^, respectively, was applied. Moreover, a 44 cm metal tube placed 2 cm above the burner (Burner–Tube Distance (BTD) = 2 cm) was used to enclose the flame compartment from the surrounding environment, prolonging the HTPRT and preventing cooling of the flame. Finally, the particle collection was assisted by an additional 10 L min^−1^ O_2_ sheath flow and a vacuum pump (BUSCH V40). The powder was then retrieved by scrubbing it off from a glass microfiber filter with a binder (Albet Labscience, Hahnemuehle, Dasen, Germany, GF 6 257) filter. 

### 2.2. Structural Characterization Techniques 

*Raman spectroscopy*: The structural features of the LTO NPs were evaluated by Raman spectroscopy using a HORIBA-Xplora Plus spectrometer (Kyoto, Japan), equipped with an Olympus BX41 microscope (Breinigsville, PA, USA). A 785 nm diode laser served as the excitation source, with the laser beam focused on the sample using the microscope. Raman spectra were obtained by performing 30 accumulations over 10 se at a consistent laser intensity, which was 100% of the laser’s total intensity. 

*Ultraviolet–Visible Diffuse Reflectance (UV–Vis DRS):* DRS experiments were conducted using a Perkin Elmer Lambda-35 spectrophotometer (Waltham, MA, USA) using BaSO_4_ powder as the background standard while operating at room temperature. The optical energy gap value (*E_g_*) of each LTO semiconductor was determined through extrapolation of a straight line on the curve that was formed from the correlation between (αhv)^1/n^ and hv, called the Tauc plot method [28], and the following equation:

(1) $${\left(ahv\right)}^{1/n}=C\left(hv-E_{g}\right)$$

where α is the absorption coefficient, *hv* is the photon energy, *E_g_* is the energy gap and *C* is a constant. The exponent denotes the nature of the electronic transition; when *n* = 1/2, it is a direct allowed transition, and when *n* = 2, it is an indirect allowed transition. 

*Powder X-ray Diffraction (pXRD):* In order to obtain the crystal structures of the nanoparticles, a D8 Advance Bruker diffractometer with a Cu source (Kα, λ = 1.5418 Å) and a secondary beam monochromator was used. All XRD patterns were collected at room temperature, operating at 36 kV and 36 mA. Using the Scherrer equation, the crystal size (*d_XRD_*) of the nanoparticles was calculated [29]:

(2) $$d_{XRD}=\frac{K\;\lambda }{\left(FWHM\right)*cos\theta }$$

where *K* = 0.9, *λ* = 1.5418 Å and *FWHM* is the full width at half-maximum of the XRD peaks. 

*Transmission Electron Microscopy (TEM)*: The morphology and nanostructure of the nanoparticles were unveiled using an FEI Titan 80-300 S/TEM microscope at 300 kV accelerating voltage and a 21.5 mrad beam convergence angle. The powdered sample preparation involved sonicating the samples in ethanol, followed by depositing the resulting homogeneous suspension as a single droplet onto a TEM copper grid covered with lacey carbon film.

### 2.3. Photocatalytic Evaluation

*Photocatalytic H_2_ production from H_2_O*: Photocatalytic experiments were conducted in a double-walled photochemical reactor (Toption instrument Co., Ltd. (TaiBai Road, YanTa District, Xi’an, China)) with a total volume of 340 mL at room temperature (25 °C), regulated by tap circulation. A 250 W mercury lamp (UV irradiation) was used as the light source, positioned at the geometric center of the photoreactor within a quartz immersion well. The irradiation power, measured in situ with a power meter at an average experimental distance of 3 cm, was 0.34 W cm^−2^. For each experiment, 69 mg of the catalyst was suspended in 275 mL of water/methanol mixture (methanol 20% *v*/*v*), where methanol served as a hole scavenger. To increase the photocatalytic production, 1% *w*/*w* Pt was photodeposited in situ in the reaction mixture. As the Pt precursor, dihydrogen hexachloroplatinate (IV) hydrate complex (H_2_Pt_4_C_l6_. 6H_2_O, 99.99%, Alfa Aesar (Ward Hill, MA, USA)) was used. In order to identify and quantify the produced gases, such as H_2_, a Gas Chromatography system equipped with a Thermal Conductivity Detector (TCD) (Shimadzu GC-2014, Agilent Carboxen 1000 column (Stevens Creek Blvd., Santa Clara, CA, USA), Ar carrier gas) was employed. 

## 3. Results

**Figure 2:**
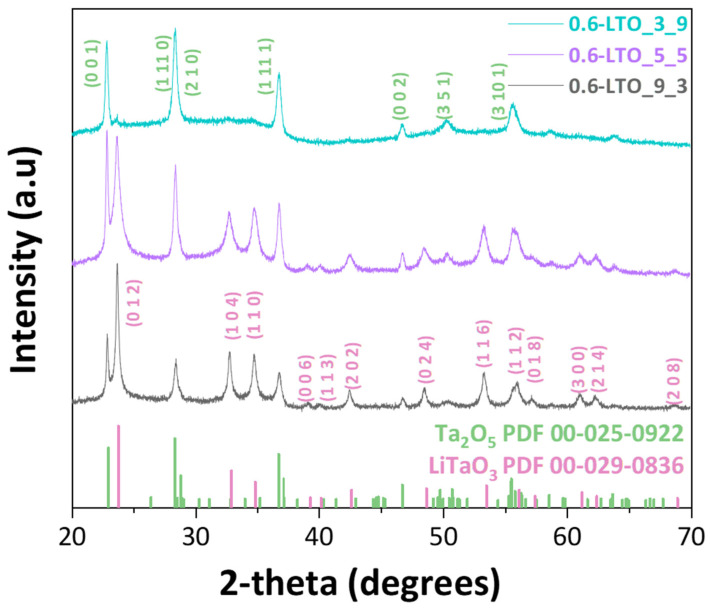
XRD patterns of the as-prepared FSP LTO nano-perovskites.

As shown in the XRD patterns in Figure 2 the diffraction peaks of the as-prepared LTO nanoparticles matched the structures of Ta_2_O_5_ (PDF 00-025-0922) and LiTaO_3_ (PDF 00-029-0836) and exhibited different phase percentages. A detailed list of the so-produced nanomaterials can be found in Table 1, where the materials are codenamed with respect to their total {Li + Ta} concentration, i.e., 0.6 M, and their corresponding P/D ratio. In all cases, the size of the crystalline domains (d*_XRD_*) was estimated from the XRD patterns using the Scherrer equation and the peaks at 22.8° and 23.8°, which correspond to (0 0 1) and (0 1 2) of Ta_2_O_5_ and LiTaO_3_, respectively; these are listed in Table 1. As such, d_XRD_ refers to the crystallite nanosize, while the size of the NPs can be further characterized using TEM.

With both the {Li + Ta} concentration and the BTD kept constant, adjusting the P/D ratio from 3/9 to 5/5 and finally to 9/3 led to an increase in the LiTaO_3_ phase percentage at the expense of Ta_2_O_5_; see Table 1. This can be understood by taking into account that an increased P/D ratio means that more precursor is injected into/combusted in the flame; this results in longer and hotter flames and an enhanced HTPRT, which ultimately defines particle growth [30]. On the opposite side, a decrease in the P/D ratio leads to shorter flames, limiting the HTPRT. In the present case, for {0.6-LTO_3_9}, the P/D = 3/9 flame resulted in 94% Ta_2_O_5_ with only 6% LiTaO_3_. As we show in Figure S1 and Table S1, in the {0.6-LTO_3_9} material, a short post-FSP treatment (480 °C, 30 min) boosted the LiTaO_3_ formation to 74%. This demonstrates that in the as-prepared {0.6-LTO_3_9}, the Li atoms are present but not incorporated into any crystalline lattice; i.e., this material is [amorphous Li] + [Ta_2_O_5_] with little LiTaO_3_. The short HTPRT for P/D = 3/9 did not allow the incorporation of Li into Ta_2_O_5_ to form LiTaO_3_.

In general, the production of ABO_3_ perovskites involves rather challenging thermodynamically driven ABO_3_ phase formation, if we take into account the competing processes: the possibility of forming separate AO_n_ and BO_m_ oxides. Moreover, in the FSP process, this should be considered taking into account the speed of the process, i.e., milliseconds of exposure time for the particles in the high-temperature region [21] and temperature gradients ranging from ~3000 °C to 600 °C at the flame’s apex. In our case, the XRD data clearly showcase the dominant role of the flame profile (via the P/D) in LiTaO_3_ perovskite formation. An increase in the P/D ratio has a profound effect on the phase composition. At P/D = 3/9, the incorporation of Li in the nanolattice failed, since we observed only the formation of Ta_2_O_5_. At a higher P/D ratio, LiTaO_3_ was formed at a percentage of 70% in the case of 0.6-LTO_9_3 due to the prolongation of the time window in which the particles remained in the high-temperature region. However, we noticed that while the crystallinity of the final material increased as the P/D ratio increased from 22.6% up to 70.6%, the percentage of the LiTaO_3_ phase had not yet reached a maximum (please see Table 1). 

Thus, the present data for LiTaO_3_, as well as our recent work on NaTaO_3_ [28], clearly exemplify that in such ABO_3_ perovskites, the P/D ratio serves a dual purpose, affecting both the crystallinity and the phase composition. However, further optimization was required for LiTaO_3_. 

To examine whether Li was present, and had not yet formed a crystal structure after FSP, the FSP process was followed by annealing at 480 °C for 30 min in atmospheric air. As shown in Figure S1, a quantitative analysis of the XRD patterns exhibited an increase in the LiTaO_3_ percentages; therefore, we can conclude that even if the temperatures inside the flame region are more than enough for the formation of the LiTaO_3_ phase, the effect of the short time-window of the FSP process is overwhelming, suppressing the formation of the perovskite structure. 

In our endeavor to produce pure-phase LiTaO_3_, we further adjusted the BTD parameter to regulate the air entrainment (AE). Process-wise, Pratsinis et al. quantitatively analyzed the role of AE during aerosol synthesis by computational fluid dynamics (CFD) and calculated the temperatures inside an enclosed FSP reactor [31]. Based on their findings, the overall temperature inside the tube decreases as AE increases due to cold gas (air) intrusion, plus dilution of the combustibles [32]. In the absence of AE, such as in a hermetically enclosed FSP burner, a recirculation vortex is expected to be formed [32], which leads to an extension of the high-temperature zone inside the reactor; therefore, the particles remain at high temperatures for a longer duration, which results in particle growth. In the intermediate case of AE = 50–100 L min^−1^, the vortex is diminished and the overall temperature inside the tube decreases, leading to reduced particle growth. For higher AE and a longer BTD, the flow and temperature patterns are similar to those for the open FSP configuration [31]. 

**Figure 3:**
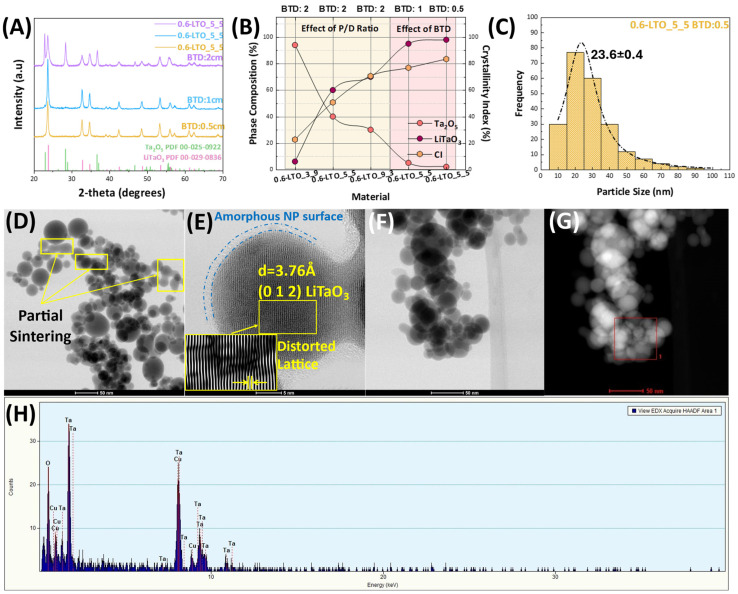
(**A**) XRD patterns of the FSP optimization process. Variation of the BTD parameter allows for control of the Ta_2_O_5_/LiTaO_3_ phase balance. (**B**) Phase composition and crystallinity index percentages of the as-prepared LTO materials. (**C**) Size distribution graph obtained from several TEM images for 0.6-LTO_5_5, BTD: 0.5. (**D**,**E**) TEM images of the as-prepared 0.6-LTO_5_5, BTD: 0.5, where we observe partial sintering due to the HTPRT. Moreover, we have the formation of a <2 nm amorphous LiTaO_3_ layer and distortions of the lattice. (**F**,**G**) STEM images and (**H**) EDX spectrum of the as-prepared 0.6-LTO_5_5, BTD: 0.5.

In our case, the formation of high-purity LiTaO_3_ nanopowders is closely related to the BTD parameter, in addition to the P/D ratio. In all cases, the {Li + Ta} concentration, HTPRT and P/D ratio were kept constant and a second series of LTO materials was prepared by adjusting only the BTD (see Figure 3A). By decreasing the BTD to 1 cm, with lower AE, we observed a dramatic effect on the Ta_2_O_5_ phase, which was reduced to 5% from 40%, while the LiTaO_3_ phase increased from 60% to 95%. A further decrease in the BTD induced small changes in the phase composition, reaching 2% and 98% for Ta_2_O_5_ and LiTaO_3_, respectively (see Figure 3B). Based on the CFD analysis by Pratsinis [31] and the research by Waser [32] regarding the effect of AE, altered via lifting-off the enclosing tube, during CuO synthesis, a clear correlation between the BTD and AE emerges. In the case of CuO, enclosure of the FSP reactor (AE = 0 L min^−1^) resulted in larger nanoparticles i.e. due to their prolonged exposure to high temperatures [33]. Increasing the BTD results in higher AE flows, smaller nanoparticles, and reduced vortex recirculation inside the tube, which subsequently leads to a decrease in the temperature flow field [31,32]. In the present case of LiTaO_3_, Figure 3A,B show that this increase in the temperature flow-field inside the FSP reactor, as induced by the smaller BTD values, resulted in NPs with higher LiTaO_3_ phase percentages. Thus, in this way we achieved the synthesis of the desired LiTaO_3_ perovskite phase in a single step, eliminating the need for post-FSP treatment. 

An analysis of TEM images using open-access software ImageJ v.1.52a, showed a size distribution ranging from 10 to 90 nm (Figure 3C), where the majority of the particles fell between 10 and 40 nm, with a mean size of *d*_TEM_ = 23.6 ± 0.4 nm. Moreover, TEM images (Figure 3D) of the highest-percentage LTO material, i.e., 0.6-LTO_5_5, BTD: 0.5, depicted the formation of quasi-spherical LiTaO_3_ particles forming neck-sintered aggregates, typical of FSP-produced nanoparticles. Additionally, Figure 3E shows the formation of crystalline Miller planes with spacing of *d*_012_ = 3.76 Å corresponding to the LiTaO_3_ phase. Energy-dispersive X-ray (EDX) analysis confirmed the existence of Ta but did not detect Li due to its low energy of characteristic radiation (Figure 3H). 

**Figure 4:**
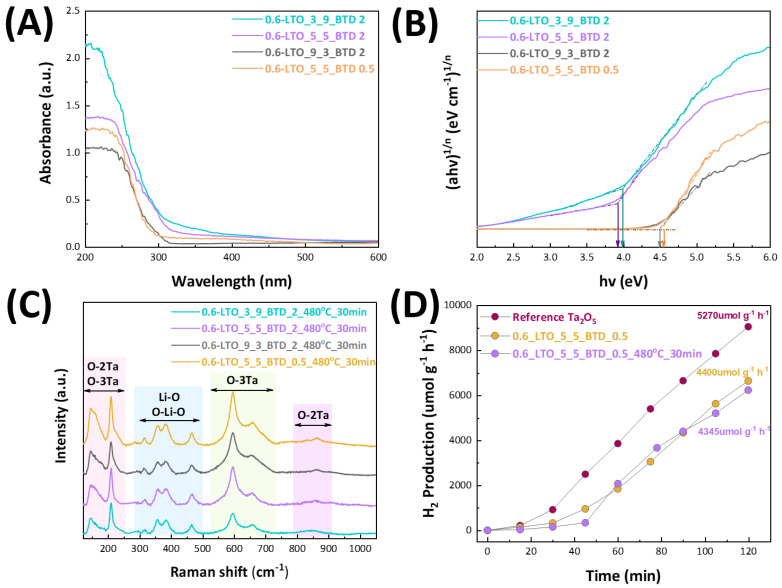
(**A**) UV–Vis DRS absorption spectra of LTO NPs. (**B**) Tauc plots derived from UV–Vis DRS spectra for LTO nanomaterials with the arrows showing the calculated *E_g_* values. (**C**) Raman spectra of the LTO nanomaterials after calcination at 480 °C for 30 min with the assigned deformation and stretching vibrations of O-2Ta and O-3Ta marked with pink, green and violet color, as well as the stretching and bending of the Li-O and O-Li-O modes with blue color, respectively. (**D**) Photocatalytic H_2_ production under UV light irradiation of 0.6_LTO_5_5_BTD_0.5 material, which possessed the highest LiTaO_3_ percentage with respect to Ta_2_O_5_.

The LTO materials were further studied using UV–Vis DRS and Raman spectroscopy. The energy gap values (*E_g_*) of the materials were estimated via the Tauc plot (see Figure 4A,B), using the Kubelka–Munk method. As anticipated, none of the samples exhibited absorption in the visible region. Their spectra consisted of a single absorption band below 400 nm, which is characteristic of this group of oxides. From the Tauc plot in Figure 4B, it is evident that materials 0.6-LTO_3_9_BTD_2 and 0.6-LTO_5_5_BTD_2 exhibited *E_g_* values of 4 eV and 3.92 eV, respectively. These values are in accordance with literature values for Ta_2_O_5_ *E_g_* equal to 4 eV [33]. At P/D = 9/3, where the LiTaO_3_ percentage was higher, we observed an increment in *E_g_* up to 4.5 eV, a value close to the literature *E_g_* of 4.7–4.84 eV for LiTaO_3_ [10,11].

Figure S2 in the Supporting Information shows the Raman spectra of the as-prepared LTO materials. In all cases, the absence of well-defined Raman peaks witnesses the formation of an amorphous/semi-crystalline surface on the LTO flame-made perovskites, which is also evident from the TEM images (see Figure 3E). Post-FSP annealing at 480 °C for 30 min of the LTO materials produced Raman spectra of enhanced intensity and sharpness, shown in Figure 4C. According to the literature [34,35], the Raman bands at <250 cm^−1^ are attributed to the internal deformation of O-2Ta and O-3Ta, while the bands between 300 and 500 cm^−1^ correspond to Li-O stretching and O-Li-O bending modes. Furthermore, the bands shown at the regions between 550 and 1000 cm^−1^ are ascribed to the stretching modes of O-3Ta and O-2Ta [34,35]. Overall, taking into account the XRD and Raman spectra, the data in Table 2 allow us to safely conclude that, in these cases, Li atoms were successfully incorporated into the Ta_2_O_5_ lattice to form a stable LiTaO_3_ lattice. 

Figure 4D shows the photocatalytic activity of the as-prepared and calcined materials, with the highest percentage of LiTaO_3_, i.e., 0.6_LTO_5_5_BTD_0.5 and 0.6_LTO_5_5_BTD_0.5_480 °C_30 min, respectively. Compared to the photocatalytic activity of pristine Ta_2_O_5_, both LTO perovskites exhibited lower H_2_ production under UV irradiation i.e. despite the improved crystallinity induced by the refinement of the BTD. In all cases, Pt^0^ was used as a co-catalyst due to its high work function of Φ_Pt_ = 5.65 eV, compared to those of other noble metal co-catalysts (Φ_Pd_ = 5.30 eV, Φ_Au_ = 5.21 eV and Φ_Ag_ = 4.26 eV) [36], which leads to a high energy difference between the conduction band of the semiconductor and the noble metal co-catalyst. This energy difference favors the formation of a stronger Schottky barrier and the trapping of electrons in the conduction band of the semiconductor. 

Moreover, the slow electron accumulation by the cocatalyst, in the case of LTO materials, leads to retardation of the H_2_ production rate, up to 45 min, compared to 15 min delay, observed with Ta_2_O_5_. However, these results do not come as a surprise, due to the difference in the energy gap values of Ta_2_O_5_ and LiTaO_3_, as we have already stated. Besides this, all ATaO_3_ compounds (A being Li, Na or K) feature corner-sharing TaO_6_ octahedra forming a perovskite-like structure. The photocatalytic activities of these materials depend on the A-site cation of the structure. It was also reported that the Ta-O-Ta bond angles have an intrinsic role in photocatalytic activity and aid in energy delocalization [37,38]. LiTaO_3_ exhibits a bond angle of 143°, while NaTaO_3_ and KTaO_3_ form bond angles of 163° and 180°, respectively. This makes LiTaO_3_ a tantalate perovskite with high *E_g_*, high distortions and low energy delocalization compared to the aforementioned Ta-based materials. 

## 4. Conclusions

In the present work, we extended the versatility of FSP in the family of Ta perovskites through the successful synthesis of LiTaO_3_. By adjusting the P/D ratio, which affects both the combustion enthalpy and the HTPRT, simultaneous crystallization and phase transformation from {amorphous Li/Ta_2_O_5_} to {Ta_2_O_5_-LiTaO_3_} was achieved. However, post-calcination of the flame-made Ta_2_O_5_-LiTaO_3_ sample enhanced the LiTaO_3_ phase percentage. Further optimization involved control of the AE by adjusting the BTD, which affected the overall HTPRT profile. These findings imply that the flame synthesis of LiTaO_3_ requires finetuning of the exposure of the NPs to the HTPRT zone to achieve high crystallinity and the incorporation of Li into the Ta_2_O_5_ nanolattice. 

However, despite the bold advancements in FSP-made perovskite synthesis, some critical problems emerge. Technology-wise, lithium and tantalum are considered critical elements in Europe due to their vital roles in emerging technologies and their limited supply. Lithium is a key component in rechargeable batteries, particularly for electric vehicles and renewable energy storage systems, which are vital for the transition to a low-carbon economy. Tantalum is widely used in electronic components, such as capacitors and semiconductors, which are crucial for modern electronics and communication systems. Intensive use of Li and Ta will lead to resource scarcity, environmental impacts, supply chain vulnerabilities and economic pressures. To mitigate these challenges, there will be an increased need for technological innovation. This includes developing alternative materials, advancing recycling technologies and improving the efficiency and lifespan of products that utilize lithium and tantalum. 

## Figures and Tables

**Table 1:** FSP process parameters and structural characteristics of the as-prepared LiTaO_3_ NPs.

Nanomaterial	P/D	[Li/Ta] Concentration (mM)	BTD (cm)	HTPRT (cm)	% Phase (±5%)		Crystallite Size (nm) (±0.5 nm) ^1^		CI (%) ^2^
					Ta_2_O_5_ (%)	LiTaO_3_ (%)	*d*_XRD_ Ta_2_O_5_	*d*_XRD_ LiTaO_3_	
0.6-LTO_3_9	3/9	300:300	2	44	94	6	33.8	-	22.6
0.6-LTO_5_5	5/5	300:300	2	44	40	60	26.7	16.6	50.7
0.6-LTO_9_3	9/3	300:300	2	44	30	70	24	27.9	70.6
0.6-LTO_5_5	5/5	300:300	1	44	5	95	-	15.2	76.8
0.6-LTO_5_5	5/5	300:300	0.5	44	2	98	-	25	83.4

^1^ The ±0.5 nm uncertainty stems from the uncertainty in the estimations of the FWHM of the XRD peaks. ^2^ Crystallinity index (CI) = 100% − amorphous content (%). The amorphous content was determined by measuring the area under the curvature hump in the XRD baseline.

**Table 2:** Summarized Raman band frequencies of the calcined materials and the vibrational modes in the crystal lattice that correspond to these frequencies.

Phonon Branch	Assignment	Raman Shift (cm^−1^)	Raman Shift (cm^−1^) (Literature)
E (TO_1_)	O-3Ta and O-2Ta deformation	145	143 [34], 144 [35]
A_1_ (TO_1_)		209	208 [34], 209 [35]
E (TO_4_)	Li-O stretching and O-Li-O bending	317	315 [34,35]
A_1_ (LO_2_)		357	356 [34,35]
E (LO_5_)		381	383 [34], 380 [35]
E (TO_7_)		466	465 [34], 463 [35]
A_1_ (TO_4_)	O-3Ta stretching	597	596 [34,35]
E (LO_8_)		659	661 [34,35]
A_1_ (LO_4_)	O-2Ta stretching	863	861 [34], 863 [35]

## Data Availability

Data is contained within the article or Supplementary Materials.

## Supplementary Materials

The following supporting information can be downloaded at: https://www.mdpi.com/article/10.3390/nano14151257/s1, Figure S1: XRD patterns of the post-FSP treated materials; Table S1: Structural characteristics of the post-FSP treated materials; Figure S2: Raman spectra of the as-prepared LTO materials.
