# Supplementary material for: Engineering of LiTaO3 Nanoparticles by Flame Spray Pyrolysis: Understanding In Situ Li-Incorporation into the Ta2O5 Lattice
Source: Nanomaterials (Basel). 2024 Jul 27;14(15):1257. doi: 10.3390/nano14151257 (PMC11314277; doi:10.3390/nano14151257)
Supplement: Supplementary file 1 [file nanomaterials-14-01257-s001.zip › nanomaterials-3108582-supplementary.pdf]

Supplementary Materials

Engineering of $LiTaO_3$ Nanoparticles by Flame Spray Pyrolysis: Understanding the In Situ Li-Incorporation into the Ta_2O_5 Lattice

Pavlos Psathas, Areti Zindrou, Anastasia V. Spyrou and Yiannis Deligiannakis*

Laboratory of Physical Chemistry of Materials & Environment, Department of Physics, University of Ioannina, 45110 Ioannina, Greece

* Correspondence: ideligia@uoi.gr; Tel.: +302651008662

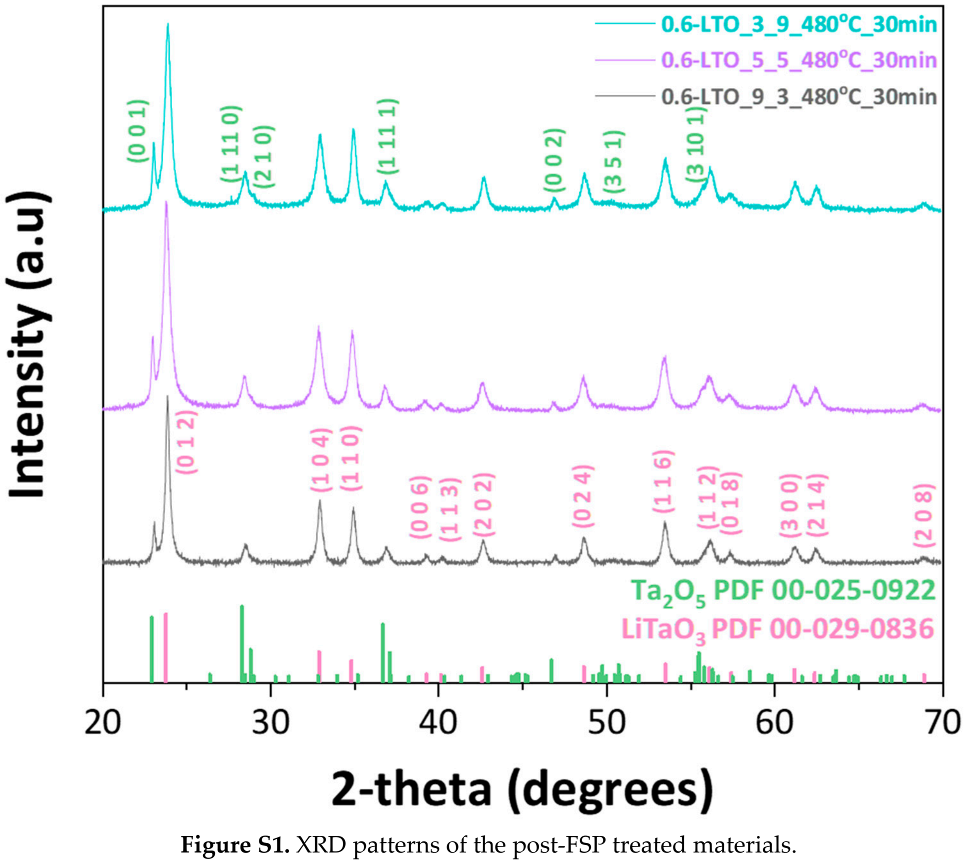

Figure S1. XRD patterns of the post-FSP treated materials.

Table S1: Structural characteristics of the post-FSP treated materials.

Nanomaterial	% phase (± 5%)		Crystallite size (nm) (±0.5nm)	
	Ta_2O_5 (%)	$LiTaO_3$ (%)	d_{XRD} Ta_2O_5	d_{XRD} $LiTaO_3$
0.6-LTO_3_9_480°C_30min	26	74	18.6	20.6
0.6-LTO_5_5_480°C_30min	22	78	22.5	18.8
0.6-LTO_9_3_480°C_30min	88	12	23.2	30.3

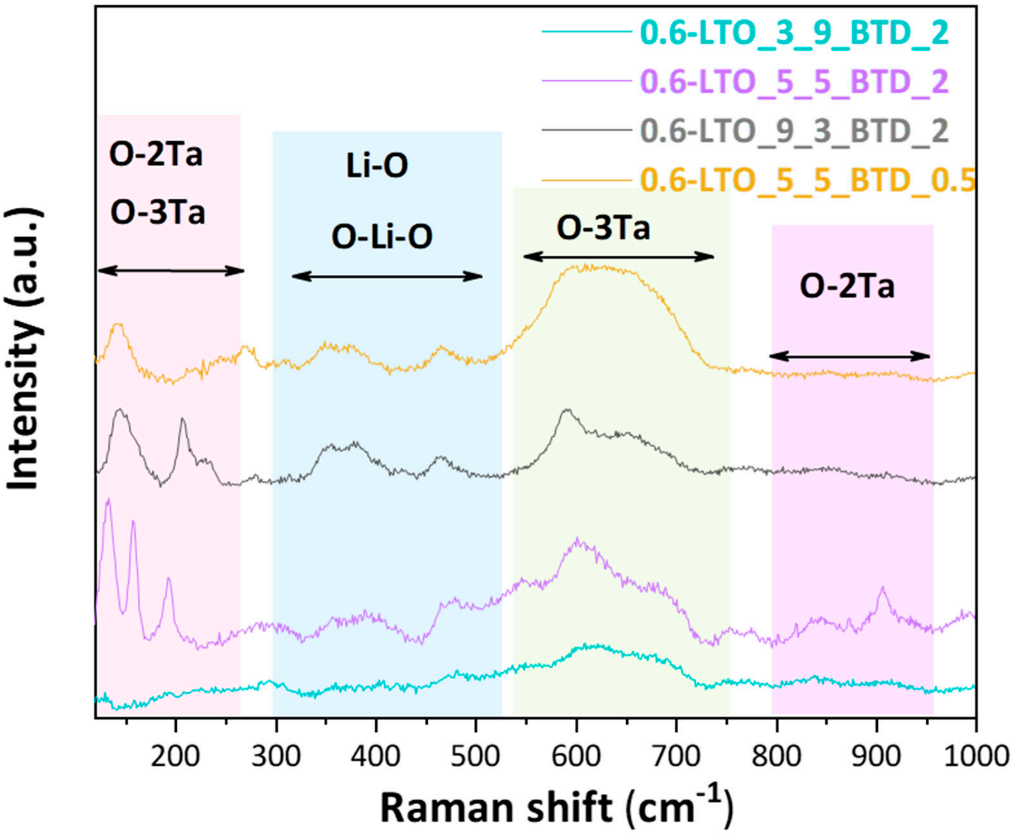

Figure S2. Raman spectra of the as prepared LTO materials.
